# Listening to Women: Experiences of Using Closed-Loop in Type 1 Diabetes Pregnancy

**DOI:** 10.1089/dia.2023.0323

**Published:** 2023-11-23

**Authors:** Julia Lawton, Barbara Kimbell, Mia Closs, Sara Hartnell, Tara T.M. Lee, Anna R. Dover, Rebecca M. Reynolds, Corinne Collett, Katharine Barnard-Kelly, Roman Hovorka, David Rankin, Helen R. Murphy

**Affiliations:** ^1^ Usher Institute, Medical School, University of Edinburgh, Edinburgh, United Kingdom.; ^2^ Cambridge University Hospitals NHS Foundation Trust, Wolfson Diabetes and Endocrine Clinic, Cambridge, United Kingdom.; ^3^ Norwich Medical School, Floor 2, Bob Champion Research and Education Building, James Watson Road, University of East Anglia, Norwich Research Park, Norwich, United Kingdom.; ^4^ Norfolk & Norwich University Hospital NHS Foundation Trust, Norwich, United Kingdom.; ^5^ Edinburgh Centre for Endocrinology and Diabetes, Royal Infirmary of Edinburgh, Edinburgh, United Kingdom.; ^6^ Centre for Cardiovascular Science, Queen's Medical Research Institute, University of Edinburgh, Edinburgh, United Kingdom.; ^7^ Norwich Clinical Trials Unit, Norwich Medical School, University of East Anglia, Norwich, United Kingdom.; ^8^ Spotlight-AQ limited, Southern Health NHS Foundation Trust, Southampton, United Kingdom.; ^9^ Wellcome Trust-MRC Institute of Metabolic Science, University of Cambridge, Cambridge, United Kingdom.; ^10^ Department of Paediatrics, University of Cambridge, Cambridge, United Kingdom.

**Keywords:** Closed-loop, Pregnancy, Type I diabetes, Women's experiences, Quality-of-life

## Abstract

**Introduction:**

Recent high-profile calls have emphasized that women's experiences should be considered in maternity care provisioning. We explored women's experiences of using closed-loop during type 1 diabetes (T1D) pregnancy to inform decision-making about antenatal rollout and guidance and support given to future users.

**Methods:**

We interviewed 23 closed-loop participants in the Automated insulin Delivery Among Pregnant women with T1D (AiDAPT) trial after randomization to closed-loop and ∼20 weeks later. Data were analyzed thematically.

**Results:**

Women described how closed-loop lessened the physical and mental demands of diabetes management, enabling them to feel more normal and sleep better. By virtue of spending increased time-in-range, women also worried less about risks to their baby and being judged negatively by health care professionals. Most noted that intensive input and support during early pregnancy had been crucial to adjusting to, and developing confidence in, the technology. Women emphasized that attaining pregnancy glucose targets still required ongoing effort from themselves and the health care team. Women described needing education to help them determine when, and how, to intervene and when to allow the closed-loop to operate without interference. All women reported more enjoyable pregnancy experiences as a result of using closed-loop; some also noted being able to remain longer in paid employment.

**Conclusions:**

Study findings endorse closed-loop use in T1D pregnancy by highlighting how the technology can facilitate positive pregnancy experiences. To realize fully the benefits of closed-loop, pregnant women would benefit from initial intensive oversight and support together with closed-loop specific education and training.

Clinical Trial Registration number: NCT04938557.

## Introduction

To reduce risks of obstetric and neonatal complications, pregnant women with type 1 diabetes (T1D) are advised to keep glucose between 3.5 and 7.8 mmol/L [63–140.4 mg/dL] for ≥70% of the time.^1^ Women are acutely aware of the risks T1D poses to their babies and highly motivated to address them.^2,3^ However, pregnancy-related physiological changes (e.g., nausea and vomiting, variations in insulin sensitivity and/or resistance) and limitations of subcutaneous insulin regimens can make attainment of pregnancy glucose targets extremely challenging.^4,5^

Moreover, the pressure to attain pregnancy glucose targets can cause significant psychological distress.^2,6^ Throughout pregnancy, women with T1D receive intensive clinical support (weekly/fortnightly contacts) to assess maternal glucose levels and optimize insulin doses. However, this “medicalization” can further undermine women's pregnancy enjoyment.^7^

Continuous glucose monitoring (CGM) has been shown to improve glucose levels and newborn health outcomes^8,9^ and is now widely offered in T1D pregnancy in the United Kingdom and internationally.^10^ Closed-loop systems, which link an insulin pump and CGM through a control algorithm that automates basal insulin delivery, have potential to provide additional glycemic benefits, with early reports showing promising biomedical results.^11–15^ Studies reporting pregnant women's experiences of using closed-loop technology are extremely limited and investigated early generation prototype devices used for short durations.^16,17^ Hence, their findings have limited relevance for decision-making about closed-loop use in pregnancy.

In response to high-profile calls to listen to women and take their experiences seriously in maternity care provisioning,^18^ and concerns about inadequacies in existing quality-of-life measures for pregnant women with T1D,^19^ we conducted longitudinal interviews with women who used the first commercially available closed-loop (CamAPS FX) system licensed for use in T1D pregnancy. The purpose of these interviews was to explore how women used the system, and how closed-loop use affected their diabetes management and pregnancy experiences. Our objective was to allow women's experiences to inform decision-making about antenatal closed-loop rollout and guidance and support given to future closed-loop users.

## Methods

### Overview

We interviewed women randomized to the closed-loop arm of the Automated insulin Delivery Among Pregnant women with T1D (AiDAPT) trial, which is a UK-based open-label multicenter randomized trial comparing closed-loop with standard insulin delivery.^20^ Participants used the CamAPS FX system for ∼24 weeks during their T1D pregnancy (∼13–37 weeks). The CamAPS FX app included functions enabling users to input mealtime boluses, personalize their glucose targets and increase (“Boost”) or reduce (“Ease-off”) basal insulin delivery by ∼33%.

Users could initiate and specify a start time and duration (≤12 h) for “Boost” when they felt more insulin was needed (e.g., during periods of inactivity, increased food intake, illness or stress) or “Ease-off” when less insulin was needed (e.g., during early pregnancy, exercise, or when nausea, vomiting or decreased food intake occurred). The app facilitated automatic data upload to the cloud, enabling data sharing with health care professionals (HCPs).

Further details about the trial (including eligibility criteria) and the CamAPS FX system are provided elsewhere^20^ and in Table 1. Ethics and governance approvals were obtained as part of the main trial (Cambridge Central Research Ethics Committee: 18/EE/0084). Our approach to reporting follows Standards for Reporting Qualitative Research (SRQR).^21^

**Table 1.** Information About the Trial, Devices Used, and Data Sharing

The AiDAPT trial
The AiDAPT trial (ISRCTN: 56898625) was conducted at nine maternity clinics across England, Scotland, and Northern Ireland (Norwich, Cambridge, Ipswich, Glasgow, London (Kings College Hospital, Guys and St Thomas' Hospital), Edinburgh, Leeds, and Belfast). One hundred twenty-four women were recruited. To be eligible, women had to be aged 18–45 years, have lived with T1D for at least 1 year, have a viable pregnancy confirmed by ultrasound (up to 13 weeks and 6 days gestation), been using intensive insulin therapy (MDI or an insulin pump) and have an HbA1c of 48 to ≤86 mmol/mol (6.5% to ≤10.0%). For further details about inclusion/exclusion criteria, see Lee et al.^20^
Women were randomized to use either the CamAPS FX closed-loop (intervention arm) or standard insulin delivery with CGM (control arm). Participants were asked to contact their local study team for any problems related to diabetes management. They also had access to a 24-h telephone helpline with a research educator to seek technical support.
The CamAPS FX hybrid closed-loop system
The CamAPS FX is a “hybrid” closed-loop system calculating and delivering basal (background) insulin automatically, which requires the user to administer boluses to cover meals/food. The system comprised:
• Dana RS insulin pump (Sooil, Seoul, South Korea).
• Dexcom G6 real-time CGM sensor (Dexcom, San Diego, CA, USA).
• An unlocked Android smartphone (Galaxy S7-10, Samsung, South Korea) running Android 8 OS or above, which hosted the CamAPS FX app incorporating the Cambridge model predictive control algorithm (CamDiab, Cambridge, United Kingdom) and communicating wirelessly with the insulin pump. Participants could opt to use their personal smartphone if compatible.
CamAPS FX app
In addition to being used to administer mealtime boluses, the CamAPS FX app includes functions enabling users to:
(1) Insulin delivery, insulin boluses and carbohydrate intake, high/low glucose range, glucose trend arrows, “Boost” and “Ease-off” status, and system status (operational or interrupted/switched off).
(2) View summary statistics for daily, weekly, monthly, or 3-monthly periods, including: mean CGM glucose, GMI or estimated HbA1c, time in/below/above target glucose range, number and average duration of hypos, total daily dose/bolus/basal insulin, and percentage of time in operation.
(3) Adjust the rate of insulin delivery using a “Boost” or “Ease-off” mode of operation.
(4) Set personal glucose targets, typically 5.5 mmol/L (99 mg/dL) in early pregnancy and 5.0 mmol/L (90 mg/dL) after 14–16 weeks consistent with achieving pregnancy glucose targets.
(5) Receive and personalize alarms (audio and vibration settings) triggered by high/low-glucose levels and signal loss with the sensor and/or pump.
Data sharing/remote monitoring capabilities The app automatically facilitates data upload to the cloud, which enables data sharing with other individuals, including health care teams. Clinical and research teams could view a woman's data through the Glooko/Diasend mobile app or the Diasend web application (Glooko/Diasend; Göteborg, Sweden). Health care teams had remote access to real-time data and summary statistics listed in points (1) and (2) above. HCPs could also receive summary CamAPS FX reports by e-mail, either daily, weekly, or monthly, for participants using the closed-loop at their site. These included key glycemic metrics (mean glucose; time in/above/below target glucose range), insulin doses, and system metrics (closed-loop use, CGM use, and number of alarms issued during the day and at night).

CGM, continuous glucose monitoring; GMI, Glucose Management Indicator; HCP, health care professional.

### Sampling and recruitment

Women were recruited and consented into the interview study when they consented to participate in the AiDAPT trial. Recruitment took place at seven clinical sites in England and Scotland. Purposive sampling was used to encourage diversity with respect to women's socioeconomic status, age, and parity. Recruitment continued until data saturation was reached.

### Data collection

Women were interviewed twice by a highly experienced (non-clinical) qualitative researcher (D.R.), who had no prior relationship with participants, after randomization to closed-loop and ∼20 weeks later. Baseline interviews explored women's experiences of managing diabetes during previous pregnancies (if relevant) and before using closed-loop to set the context for understanding their subsequent experiences of closed-loop use.

Interviews were informed by topic guides (see Table 2): these ensured the conversation remained relevant to addressing the study aims while allowing participants opportunities to raise issues they considered important, including those unforeseen at the outset. Topic guide development was informed by previous studies reporting user experiences of closed-loop,^22–24^ input from clinical co-investigators and revised in response to emerging findings. Interviews took place by telephone between April 2020 and April 2022, and were digitally recorded and transcribed. Interviews lasted 1–2 h.

**Table 2.** Topics Explored in Interviews of Relevance to This Analysis

Background information and pretrial experiences
• Age, occupation, living arrangements, number, and age of other children.
• Diabetes duration; devices (e.g., pump, injections, CGM, and finger pricks) used pretrial.
• Views, hopes, and concerns about managing diabetes while pregnant and related pregnancy/health impacts.
• Experiences of managing diabetes during previous pregnancies (if any) and/or current pregnancy before joining the trial, including:
○ Regimen used (insulin administration and glucose monitoring); adjustments to basal rates/background and insulin-to-carbohydrate ratios; dietary choices and managing diabetes at mealtimes; undertaking physical activity; attainment of pregnancy glucose targets.
○ Management of and worries/concerns about hypo- and hyperglycemia.
Impact of regimen used on everyday life (e.g., sleep, work/family/social life) and overall pregnancy experience.
• Experiences of and views about receiving support from HCPs during previous pregnancies and/or pre-pregnancy planning.
Experiences of using the closed-loop system during the trial
• Experiences of initial training and education, learning to use and adapting to closed-loop; developing confidence and trust in closed-loop technology; any concerns about using closed-loop (and its components) during the trial.
• Experiences of and views about using the closed-loop to manage diabetes while pregnant, including:
○ Use of the app to inform decisions about diabetes management tasks (e.g., calculating/administering mealtime bolus doses, managing/treating hypo- and hyperglycemia).
○ Use of “Boost” and “Ease-off” functions; use of corrective doses of insulin.
○ Impact of closed-loop use when physically active.
○ Ability to attain (and maintain) pregnancy glucose targets.
○ Impact of closed-loop use on worries/concerns about hypo- and hyperglycemia.
○ Engagement with and access to insulin and glucose data through app on phone (and if and how this changed over time); which of the available data participants used; if/how data access affected how they managed diabetes.
○ Perceived impact of closed-loop use on everyday life (e.g., sleep, work/family/social life)
• Impact of closed-loop use on worries/concerns about managing diabetes while pregnant and related pregnancy/health outcomes.
• Experiences of and views about contact and support received from HCPs (e.g., mode and frequency of, and reasons for, contacts; impact of closed-loop use on participants' experience of health care encounters and interactions)
• Experiences of and views about health care teams having remote access to their real-time glucose and insulin data (e.g., concerns, perceived (dis)advantages).

### Data analysis

As this work was informed by a priori as well as emergent interests, data analysis sought to identify both descriptive and analytical themes^25^ with relevance to clinical practice. Four experienced qualitative researchers (J.L., B.K., D.R., and M.C.) analyzed the data using the technique of constant comparison.^26^ First, all interview transcripts were read through repeatedly and cross-compared to identify key cross-cutting themes. Next, a coding frame was developed to capture data relevant to each of these themes.

Coded data sets were then subject to further analyses to identify subthemes and illustrative quotations. Throughout, qualitative research team members undertook independent analyses and wrote separate reports before meeting to discuss their interpretations and agree on the main findings/themes. The qualitative software package Nvivo 20 (QSR International, Doncaster, Australia) was used to facilitate data coding and retrieval.

## Results

Twenty-three women participated. See Table 3 for information about the sample, including demographic characteristics and pre-trial glucose monitoring and insulin regimens.

**Table 3.** Sample Characteristics, *n* = 23 Pregnant Women with Type 1 Diabetes

Characteristic	*n*	%*^a^*	Mean, SD, (range)
Married/cohabiting	20	87 · 0	
Employment			
Full time	10	43 · 5	
Part time	10	43.5	
Unemployed/student	1	4.3	
Full-time mother/carer	2	8 · 7	
Occupation^b^			
Managers	1	4.3	
Professionals	6	26.1	
Technicians and associate professionals	5	21.7	
Clerical support workers	1	4.3	
Service and sales workers	2	8.7	
Craft and related trades workers	1	4.3	
Elementary occupations (e.g., manual)	4	17.4	
Student/unemployed	1	4.3	
Full-time mother/carer	2	8.7	
Ethnicity			
White, British	21	91 · 3	
White, other nationality	2	8 · 7	
Age at time of interview, years			31.5 ± 4.6 (22–39)
No. of previous pregnancies			1.3 ± 1.2 (0–5)
Diabetes duration, years since diagnosis			18.6 ± 6.8 (2–28)
Baseline HbA1c			
mmol/mol			59 ± 10.6 (48–90)
%			7.5 ± 1.0 (6.5–10.4)
Devices used before current pregnancy			
Insulin regimen			
Multiple daily injections	12	52.2	
Insulin pump	11	47.8	
Self-reported glucose monitoring:			
Finger-prick testing	10^c^	43.5	
Freestyle Libre1	7	30.4	
Freestyle Libre2	2	8.7	
Dexcom-G6	4	17.4	

^a^ Figures may not add up to 100% due to rounding.

^b^ Defined using the International Standard Classification of Occupations 2008 (ISCO-08).

^c^ Seven of these women were given use of a sensor (in most cases, Freestyle Libre 1) near the start of their current pregnancy, that is, shortly before joining the AiDAPT trial.

SD, standard deviation.

We begin by reporting women's experiences of diabetes management during previous pregnancies and before using closed-loop in their current pregnancy to set the context for their subsequent closed-loop use. We then describe the perceived benefits and limitations to closed-loop use, and the importance most women placed upon collaborating both with the system and HCPs to attain pregnancy glucose targets. Finally, we present women's views about the impact of closed-loop use on their overall pregnancy experiences. Key illustrative quotations are included below; for additional quotes see Table 4.

**Table 4.** Additional Participant Quotations

Themes/subthemes	Participant quotations
Managing T1D pregnancy before closed-loop	
Physical, mental, and emotional demands	Needing to set alarms at night: “The hardest thing is at night I think, ‘cause I've quite a fear of going low. So I'd set about three alarms overnight (laughs). You just end up not sleeping very well at all and I think that can kind of get you down.” (010) “With my other pregnancy they wanted overnight readings. They wanted them at a certain time, so I'd have to set my alarm, wake myself up, test my blood, go back to sleep… it was exhausting.” (014)
Negative pregnancy experiences	Becoming obsessed about monitoring and over-correcting: “I've been using a lot of temporary basals, and if anything I was doing overcorrection sometimes. So I was finding I was, you know, I'd be hypo and then sort it out, and then I'd get a massive rebound high… So I was getting a lot of peaks and troughs, and I was finding that very stressful.” (022)
Experiences of closed-loop	
Adjusting to the system	Frequent data checking to seek reassurance: “It felt as though I was just constantly watching, making sure that it was doing its job, so I would be probably looking at it anywhere between- I would probably say six to ten times a day. I was constantly checking on it.” (019) Contacting HCPs for information and support: “It was easier to explain when you are using it, rather than as you set it up, you know, it's easy to say: oh this one means it's rising, this one means it's lowering, but it's not until I started using that that I realised I didn't actually fully understand the function and needed a bit more support.” (014) “The training was very good, it was thorough, but you will be learning as you start to use it… I've messaged [names staff member] a couple of times, initially particularly when my sugars were going high, I was like: normally I'd give a correction here, I'm going to put the Boost function on: is that right?… Shall I use it for this amount of time or longer? - So it's just that clarification.” (022)
Less work, less worry… better glucose control	Remote insulin administration facilitating more time in range: “Before… if my Libre said I was 12 [mmol/L - 216 mg/dL] and I was in the playground with lots of other mums … and I knew I was going home in half an hour, then I wouldn't get my insulin pen out to give myself a correction… especially when you're pregnant, you don't wanna get your tummy out to (laughing) give yourself an injection… whereas you can do that now. So again, that's another factor that just means your time in target must be, yeah, just hugely better.” (011) Experiencing better sleep: “I think obviously being the closed-loop, it adjusts for you…in the background… ‘cause I never really knew what my overnights were. Even with the Libre you have the Libre lows, my overnights were sort of all over the place. Whereas now I could have a steady night, and obviously sleep, and not have to worry too much about it.” (021) “It's definitely took the worry away for me, ‘cause I'm quite active in the day with my kids anyway, so if I'm dipping low and I'm busy with the kids, I'm then alerted before anything goes wrong, because if I was to, God forbid, have a hypo and not be responsive with my children, it would be awful.” (011)
But still work… user collaboration with closed-loop	Needing to create the conditions to help the technology work optimally: “I still think… a lot of it is your own doing and the information you're putting in and when. Em, so your carb counting, the time before you're gonna eat…” (017) Seeing both insulin and glucose data helps make better management decisions: “Having the visualisation of the graph, knowing that it's not delivering any insulin at the minute… I think that's really helpful to know that it's already eased off, so I probably haven't got that much insulin in me that's going to send me lower. So you know, that one jelly baby is going to bring me back up to the level.” (007) Needing to know when and how to intervene: “The main thing I've struggled with is, like, obviously before when my levels went high I would just put a correction dose in… But I've still kind of struggled to know… when I should put a correction in or whether I should just let the phone do its own thing.” (010) “I've messaged (names trial staff) a couple of times, when I was sort of- initially particularly when my sugars were going high, I was like: normally I'd give a correction here… I'm gonna put the boost function on: is that right?” (022)
Collaboration with closed-loop features: using Ease-off and Boost	Using Boost when the closed-loop is perceived as being too sluggish: “Sometimes I use [Boost] where I think the algorithm hasn't been as generous as I think it needs it to be, because that's just the algorithm still learning, because I'm extremely insulin resistant.” (022) Applying own knowledge to help prevent glucose excursions: “Basically it [closed-loop] does know what it's doing, but you've got that manual override if you need to, so I think… you definitely still need to have an element of knowing what you're doing as well, knowing… the bits that the [closed-loop]… doesn't know, so like your physical exercise, the food that you've just eaten and things like that.” (007) “I used Ease-off a lot at work, especially if I could see that my blood sugar was sitting just slightly lower and I knew that maybe I wasn't having lunch for like another two hours or something, to then just try and prevent a hypo.” (013)
Better collaboration with health care teams	Access to more detailed real-time data facilitates… better ad hoc clinical input: “I think it's a good thing that you can basically do a live feed to them, because it means that they've got up-to-date data that they can look at and very quickly change something if it needs to be changed. They're not looking at the five days prior, and you're saying well, now, you're having troubles now. And they're going: well, we can't see that data, so we can only go by what happened three days ago.” (019) … raises initial privacy concerns “It felt a bit Big Brother-ish at first, particularly when they would say: ‘oh well you had, you know, X number of carbs after 7 pm last Wednesday or something.” (015) …more personalised advice: “It's nice that somebody else can look at this data… they can see the graph of what's going on, how it's happening, how much insulin I've had, how much background insulin I've had. So just because they've got all that data, they can then tell me the exact thing that I need to do, which then sorts it out straightaway.” (020) … closer, more honest and trusting relationships with health care teams: “[It] allows me to communicate better, for them to understand better what I'm trying to say. And that communication, by being better, it builds trust… So I trust them more than if it was the opposite.” (002) “They have a little bit more trust in me, because they see my data and they see it's going well, so they understand my independency (sic), while maybe before they were a little bit more hesitating in giving me that independence.” (008)
Positive pregnancy experiences	Enjoying more normality and being able to work for longer: “Honestly, it allowed me to work. I would never be able… to work at the job that I was doing [waitressing] at all, if I didn't have the machine.” (002) “[Without the closed-loop] I wouldn't have gone out as much, and I wouldn't have done as much as what I done. I would have stopped work a lot more sooner than what I did…especially when you're self-employed, it does make a helluva lot of difference.” (018) Worrying less about their baby's development: “I didn't have that much fear for the pregnancy itself. And I think that's because of the closed-loop. So there are not that many concerns about the development of the- and the growth of the baby.” (007)

### Managing T1D pregnancy before closed-loop

#### Physical, mental, and emotional demands

Women described their experiences of glycemic management during previous pregnancies, in preparation for a planned pregnancy, and/or in the early stages of pregnancy as having been “such hard work… it's so intense” (010). Women, for instance, described having to “constantly prick my finger, constantly correct” (007) or “constantly chang[e] my basal rates, it was relentless” (005), and how their diabetes management had been “constantly at the forefront of my mind” (019).

Some described needing to set alarms at night to collect information needed to inform frequent adjustments to background insulin doses/basal rates or address worries about hypoglycemia, with resultant detrimental impacts on their sleep and well-being (Table 4). Women also noted how needing to make frequent alterations to background insulin doses/basal infusion rates had caused anxiety and heightened the mental demands of glycemic management throughout pregnancy:

I'm not very good at calculating things. So when they're throwing like new calculations at me… it made me really anxious… you know, constantly trying to figure out: oh, my numbers are doing this thing and they're doing that thing. What am I doing? (012)

#### Blunt instruments

Women noted that previous T1D management had been made more challenging by using blunt and inadequate instruments, including finger-prick glucose monitoring, which had resulted in them having limited and inadequate glucose information to inform self-management decisions: “I didn't have a sensor, so I couldn't look back on what my sugars were doing through the night. So it was literally guessing” (005). Excepting those who had been using CGM pretrial, women also noted how their glycemic management had been compromised by not having alarms alerting them to glucose excursions: “I wouldn't have gone all night before I realised I was at 15 [mmol/L - 270 mg/dL] or 17[mmol/L - 306 mg/dL] or something, and I could have taken a correction sooner” (015).

#### Negative pregnancy experiences

Most women reported how resultant difficulties keeping glucose within pregnancy/pre-pregnancy target ranges had led to feelings of anxiety and guilt, wherein, “every reading you see, you think, ‘oh my God, I'm harming the baby’” (010). Some noted having become “a bit obsessed” (017) about monitoring and over-correcting out-of-range glucose as a consequence, which heightened feelings of anxiety and distress (Table 4). Due to the physical, mental, and emotional demands of T1D management, women also described experiencing early pregnancy and/or previous pregnancies as “tiring and draining” (005), “very stressful” (013) and feeling that “it deprived me of enjoying it [pregnancy]” (002).

### Experiences of using closed-loop

#### Adjusting to the system

Most women described taking several weeks to develop confidence and trust in the system, with some reporting frequent data checking to seek reassurance that the closed-loop was working correctly (Table 4). Women noted how HCPs' intensive input (e.g., changing closed-loop settings), oversight and emotional support in the initial days of use had been critical to developing confidence in, and adjusting to, the system:

I trusted it, because… I just… knew that obviously the ladies at the hospital were monitoring quite closely to make sure that it was correct, and so they could change it sort of from day to day if needed. (021)

Women further noted that, although they had found the initial closed-loop training helpful and comprehensive, it had been necessary “to learn by doing” (017). Hence, they described valuing being able to text/call/e-mail HCPs after transitioning onto the system to seek practical guidance, refresh their understanding of the closed-loop's functions and seek reassurance they were using it correctly (Table 4).

#### Less work, less worry… better glucose control

After this adjustment period, women reported multiple ways closed-loop had helped reduce the physical, mental, and emotional demands of glycemic management in T1D pregnancy, although several emphasized that “the sensor would have helped even without closed-loop” (007). The key benefit, highlighted by all women, was the system's ability to automatically adjust basal insulin rates, with women noting how this had helped to reduce their physical and mental workloads while improving time spent in target glucose range:

Before… I was on it, like every couple of weeks I was having to keep changing all my basal rates and everything to try and keep up, whereas this just automatically does it, so it makes it much easier, it just takes a lot off you, like even the mental side of just constant viewing the data, it does all that for you. (010)

Women also welcomed being able to administer insulin through the app on their mobile phone, rather than through the pump or by injecting. As well as lessening their workloads and helping them feel more normal, some reported having administered correction doses more promptly due to this task being so easy and discrete to undertake (Table 4).

By virtue of the closed-loop automatically reducing or suspending insulin delivery when glucose levels fell and knowing that the CGM would alert them to glucose excursions, women described feeling more confident striving for pregnancy glucose targets:

It's definitely helped with the anxiety of running myself high to not go low, because I've got the alerts that tell me if it is gonna go low… And knowing that the closed-loop will be picking up if you are starting to get low, that it's going to ease off. (007)

Women also described experiencing better sleep (Table 4) and less stress and anxiety despite using tighter glucose targets, because of knowing that the closed-loop was operating in the background to help keep them safe:

It took the worry away for me, ‘cause I'm quite active in the day with my kids, so if I'm dipping low and I'm busy with the kids, I'm alerted before anything goes wrong, because if I was to have a hypo and not be responsive it would be awful. (011)

#### But still work… user collaboration with closed-loop

Most women, however, emphasized that closed-loop was not a panacea and that, to optimize time spent in glucose target range, they still needed to undertake some work:

Maybe just anecdotally I've heard…it's like an artificial pancreas. And I think that sounds just wrong. And I think it gives false hope… because for me it's still a lot of your management. (011)

Women, for instance, described needing to pay close attention to dietary choices, carbohydrate counting, and the timing of mealtime boluses to help create the conditions under which closed-loop could work optimally (Table 4). Furthermore, although a minority welcomed being able to delegate glucose management tasks to the system “and let it do its thing” (010), the majority expressed a strong motivation and perceived responsibility to work with, and alongside, the technology to help address and/or pre-empt glucose excursions.

In doing so, women emphasized how having easy access to “real-time” CGM glucose data, which included information about whether and how quickly glucose levels were rising or falling, had prompted and enabled more timely and informed action.

So I can see if my levels right now are stable, if they're going up, if they're going quickly up, if they're going down… So with that allows me to make a better decision… [for instance] I can see it's giving me more basal at this time, so I don't do a correction right now. (002)

Some also noted how having access to “real-time” insulin as well as CGM glucose data when using closed-loop had meant that they had better information to inform glucose management decisions (Table 4). To help ensure timely and appropriate interventions, women emphasized the importance of receiving clear instruction and education to help them determine whether, when, and how, they should intervene to address out-of-range readings and when they should allow the closed-loop to operate without interference (Table 4).

#### Collaboration with closed-loop technology; using Ease-off and Boost

Women described the Ease-off and Boost functions as being particularly valuable tools to support attainment of tight pregnancy glucose targets. Some, for instance, described benefiting from using Boost in situations where they felt the algorithm had been too sluggish and/or had struggled to keep up with their rapidly changing insulin requirements (Table 4). Women also valued using these features on occasions when, in conjunction with the closed-loop system's insulin adjustment capabilities, they felt that applying their own knowledge could help prevent glucose excursions. This included situations where they were about to undertake physical activity, eat a meal with a high fat-to-carbohydrate content, or delay a meal (Table 4).

#### Better collaboration with health care teams

Alongside their own collaborative role, women saw their health care team as playing a pivotal role in supporting effective closed-loop use by guiding and advising on appropriate courses of action, such as when to alter insulin-to-carbohydrate ratios. In doing so, women noted how, by having easy access to their data and, hence, being able to monitor their progress between, and in preparation for, antenatal appointments, their health care team had been able to provide more effective and timely input:

They check on me very few days and if they can see that: oh, you're going high at this point, or low at this point, they'll message me or ring me and say: ooh, would you mind tweaking this on your pump, or on your app. (003)

Some women described calling or texting HCPs between scheduled appointments and receiving better feedback because they did not need to rely on retrospective or self-reported information/data (Table 4). While some such women voiced initial (privacy) concerns about HCPs being able to access their insulin as well as glucose data when using closed-loop (Table 4), most emphasized that they had received better and more personalized input as a result of HCPs having access to data that allowed them “to know me, know my body…know what influences what” (002) (Table 4). Some further suggested that this enhanced data access had helped them develop more honest, positive, and trusting relationships with their health care team (Table 4).

#### Positive pregnancy experiences

Women emphasized that using closed-loop had had a positive impact on their pregnancy experiences. They partly attributed this to expending less time and effort on T1D management and, hence, enjoying a “more normal life” (002), which, in some cases, included feeling able to work for longer than in previous pregnancies (Table 4) and/or having more time to devote to childcare and other family activities.

As a result of spending increased time in target glucose range, women also described worrying less about their baby's development (Table 4) and feeling less anxious about attending antenatal appointments and receiving negative judgments:

I didn't want to go into the hospital and be told off… so I didn't really like going in, speaking to anybody. But now my sugars are so much better…it's not a worry for me anymore. I don't ever really have any issues. So it's a nicer experience for me. (011)

All women noted that, as a result of using closed-loop, they had been able to better enjoy their pregnancy:

In terms of enjoyment factor, and how I feel about the baby, and how I feel about the coming labour, and how excited I am to meet them and the bonding process and stuff like that, I'd say I've had a lot more time for it this time round. (016)

## Discussion

Women from diverse socioeconomic backgrounds reported wide-ranging glycemic and quality-of-life benefits to using closed-loop in pregnancy. In doing so, women emphasized that closed-loop was not a panacea and that, to optimize clinical gains, ongoing involvement and effort was required. Indeed, women's accounts suggest that closed-loop should be understood as one pillar in a three-party collaboration involving themselves, the technology, and their health care team.

Although women reported some anxieties when they first transitioned onto closed-loop, they did not describe the substantial negative psychosocial impacts highlighted in earlier studies.^16,17^ Instead, women emphasized quality-of-life gains resulting from lessened physical and mental workloads, experiencing better sleep, having more positive interactions with the health care team, and knowing that their baby was less “at risk” because closed-loop increased time in target glucose range. Arguably, these important benefits were not captured in earlier studies that reported women's experiences in the initial weeks of closed-loop use when “obsessive” data checking was more common.^16,17^ Furthermore, women in previous studies used prototype devices that were prone to anxiety-provoking technical malfunctions.^16,17^

Women described benefitting from intensive oversight and support from health care teams in the initial period of using closed-loop to receive practical/technical support and reassurance that the closed-loop was working effectively. Hence, similar initial intensive support should be provided to pregnant women using the technology in nontrial settings, a view shared by HCPs in a companion study, who suggested ways this support could be actualized, including provisioning of a 24-h helpline.^27^ Women also described gaining reassurance from knowing that health care teams had easy access to their data and reported receiving better and more timely input as a result; parents of young children who used the same (CamAPS FX) system have reported similar benefits.^23^

Women also reported experiencing more positive interactions with health care teams; in part, because they were less worried about being criticized for spending time out-of-target glucose range. Importantly, although some women raised initial concerns about their privacy, most described having more honest, positive, and effective collaborative relationships with HCPs by virtue of them having access to their insulin as well as glucose data. As others have observed, access to such data can offer insight into users' personal lives.^28^ Hence, it is important that health care teams use nonjudgmental collaborative approaches to ensure these positive trusting relationships are replicated in “real-world” settings.^28^

Unlike other user groups who (mostly) welcomed opportunities to delegate glucose management to the system,^23,24,29^ most pregnant women described wanting, and expecting, to maintain active self-management roles and, hence, needing training and skills to be able to do so. Arguably, this finding is partly due to the tighter glucose targets required in T1D pregnancy and the (perceived) moral mandate pregnant women experience to do everything possible to protect their babies.^2,3^

Indeed, women in our study emphasized the importance of receiving pregnancy-specific closed-loop education and training to help empower them to make informed responsible self-management decisions. It is vital, therefore, that users be given comprehensive T1D pregnancy advice and tailored information to support optimal closed-loop use in routine care settings. Women in our study valued opportunities to use the system's Ease-off and Boost features to attain pregnancy glucose targets. Future pregnant women with T1D would, therefore, benefit from systems that offer this kind of functionality; albeit, with appropriate training in place to help ensure they use these functions correctly.

As well as highlighting glycemic benefits that mirror main trial results; namely, that use of hybrid closed-loop can significantly improve maternal glycemia during T1D pregnancy,^30^ women described substantial quality-of-life benefits. Women, for example, reported feeling more normal as a result of using closed-loop, and being able to administer insulin discreetly through their mobile phone app; a benefit adolescent users also reported.^29^ Some pregnant women also reported that using closed-loop allowed them to remain in paid work for longer. This is very encouraging given that other studies have found that the pressures of managing diabetes in pregnancy can result in women prematurely leaving employment.^3^ Most crucially, women described how using closed-loop enabled them to worry less and enjoy their pregnancy more. This is a very important finding as adverse birth outcomes have been associated with maternal anxiety during pregnancy.^31^

Women emphasized that some of the benefits to using closed-loop were attributable to the CGM component. This included having access to better information to inform diabetes management decisions, and alarms facilitating use of tighter targets and ameliorating anxieties about hypoglycemia. Research involving other groups of CGM users has identified similar benefits.^32,33^ However, our findings are important as benefits to using CGM in pregnancy have not previously been reported. Moreover, they provide support for United Kingdom and international guideline recommendations that CGM be universally offered to all pregnant women with T1D.^10^

### Strengths and limitations

We have reported women's experiences of using the first commercially available closed-loop system licensed for use during pregnancy. In doing so, we have highlighted multiple quality-of-life benefits to using closed-loop in pregnancy, which are unlikely to be captured in questionnaire studies.^19^ Although we were able to explore pregnant women's experiences over a relatively long duration, it was not possible to explore closed-loop use during antenatal hospital admissions, labor, and birth. In addition, we did not interview women in the trial's control arm.

However, we did have access to women's accounts of managing T1D without a closed-loop in their previous pregnancies and/or before using closed-loop in their current pregnancy. Future research could directly compare accounts from pregnant women using closed-loop with those of pregnant women using other diabetes regimens. Unlike previous diabetes technology studies, our sample was not skewed toward middle-class individuals. Many interviews took place during the Covid-19 pandemic; hence, we may have captured women's perspectives when their anxiety levels were high, and this may have influenced their accounts.

## Conclusions

By showing that closed-loop use can lead to more positive and enjoyable pregnancy experiences, our findings, alongside main trial results,^30^ offer powerful endorsement for closed-loop use in T1D pregnancy and recent guidance in the United Kingdom to make this technology available to all pregnant women with T1D.^34^ However, as women's accounts powerfully highlight, closed-loop is not a panacea. To realize fully the benefits this technology can offer and support successful adoption and rollout in routine clinical care, women would benefit from initial, intensive input, oversight, and support from their health care team together with comprehensive closed-loop-specific education and training.

## Supplementary Material

Supplemental data
