# Supplementary material for: Listening to Women: Experiences of Using Closed-Loop in Type 1 Diabetes Pregnancy
Source: Diabetes Technol Ther. 2023 Nov 23;25(12):845–55. doi: 10.1089/dia.2023.0323 (PMC10698780; doi:10.1089/dia.2023.0323)
Supplement: Supplemental data [file Suppl_File.docx]

**Supplementary file: AiDAPT Collaborative Group membership**

Katharine Hunt, Helen Rogers, King’s College Hospital, London, UK.

Damian Morris, Duncan Fowler, Josephine Rosier, Zeenat Banu, Sarah Barker, Gerry Rayman, Ipswich Hospital NHS Trust, Ipswich, UK.

Eleanor Gurnell, Caroline Byrne, Andrea Lake, Katy Davenport, Jeannie Grisoni, Sara Hartnell, Shannon Savine, Cambridge University Hospitals NHS Foundation Trust, Cambridge, UK.

Helen Murphy, Tara Lee, Tara Wallace, Alastair McKelvey, Elizabeth Turner, Nina Willer, Norfolk and Norwich University Hospital, Norwich, UK.

Corinne Collett, Mei-See Man, Emma Flanagan, Matt Hammond, Lee Shepstone, Norwich Clinical Trials Unit, Norwich, UK.

Anna Brackenridge, Sara White, Anna Reid, Olanike Okolo, Guys and St Thomas’ NHS Foundation Trust, London, UK.

Eleanor Scott, Del Endersby, Leeds Teaching Hospitals NHS Trust, Leeds, UK.

Anna Dover, Frances Dougherty, Susan Johnston, Rebecca Reynolds, Royal Infirmary of Edinburgh, Edinburgh, UK.

Robert Lindsay, David Carty, Sharon Mackin, Isobel Crawford, Ross Buchan, Glasgow Royal Infirmary, Glasgow, UK.

David McCance, Helen Wallace, Louisa Dunlop, Joanne Quinn, Belfast Health and Social Care Trust, Belfast, Northern Ireland.

Sarah Cains, Goher Ayman, Patient and Public Involvement (PPI) leads.

Julia Lawton, David Rankin, Ruth Hart, Barbara Kimbell, Mia Nelson, University of Edinburgh, Edinburgh, UK.
